# Analysis of Dental Enamel Surface Submitted to Fruit Juice Plus Soymilk by Micro X-Ray Fluorescence:* In Vitro* Study

**DOI:** 10.1155/2016/8123769

**Published:** 2016-02-08

**Authors:** Janaína Salmos Brito, Alexandrino Santos Neto, Luciano Silva, Rebeca Menezes, Natália Araújo, Vanda Carneiro, Lara Magalhães Moreno, Jéssica Miranda, Pâmella Álvares, Giselle Nevares, Felipe Xavier, José Alcides Arruda, Ricardo Bessa-Nogueira, Natanael Santos, Gabriela Queiroz, Ana Paula Sobral, Márcia Silveira, Diana Albuquerque, Marleny Gerbi

**Affiliations:** ^1^Department of Operative Dentistry and Endodontics, Dental College of Pernambuco, University of Pernambuco, Avenida General Newton Cavalcante 1650, Tabatinga, 54753-020 Camaragibe, PE, Brazil; ^2^Health Education Department, Federal University of Sergipe, São Cristóvão, SE, Brazil; ^3^School of Dentistry, Federal University of Alagoas, Maceió, AL, Brazil

## Abstract

*Objective*. This paper aimed to analyze the* in vitro* industrialized fruit juices effect plus soy to establish the erosive potential of these solutions.* Materials and Methods*. Seventy bovine incisors were selected after being evaluated under stereomicroscope. Their crowns were prepared and randomly divided into 7 groups, using microhardness with allocation criteria. The crowns were submitted to the fruit juice plus soy during 15 days, twice a day. The pH values, acid titration, and Knoop microhardness were recorded and the specimens were evaluated using X-ray microfluorescence (*µ*XRF).* Results*. The pH average for all juices and after 3 days was significantly below the critical value for dental erosion. In average, the pH value decreases 14% comparing initial time and pH after 3 days. Comparing before and after, there was a 49% microhardness decrease measured in groups (*p* < 0.05). Groups G1, G2, G5, and G6 are above this average. The analysis by *μ*XRF showed a decrease of approximately 7% Ca and 4% P on bovine crowns surface. Florida (FL) statistical analysis showed a statistically significant 1 difference between groups. Thus, a tooth chance to suffer demineralization due to industrialized fruit juices plus soy is real.

## 1. Introduction

Dental erosion is a progressive loss of hard dental tissue generally caused by acids from any source other than bacteria. Thus, erosion is responsible for mineral loss; it may be regarded as one of the main precursors of dental cavities [1–3]. Thus, erosion usually starts on the enamel surface and may be a result of intrinsic factors, such as decreased salivary flow, gastroesophageal reflux (GER), or eating disorders such as anorexia and bulimia nervosa. Extrinsic factors may also play an important role in the establishment of dental erosion. Some of these factors are the diet and the use of drugs and supplements such as vitamin C and hydrochloric acid and aspirin. There are also idiopathic factors [4, 5].

Modern lifestyle considered has promoted changes in the populational habits. Most people are now ingesting a considerable amount of fruit, yogurts, fruit drink plus soy, and mainly more processed products like traditional fruit juices and sports and energy beverages or acidic drinks with low sugar content. In 1966, partly because of the success or a product named Vitasoy in Hong Kong, Coca Cola Co. started a project to develop a protein beverage for countries with nutrition problems in order to implement protein to the population in general. In 1975, the Brazilian Institute of Food Technology (ITAL) developed a soymilk brand named Vital, which was sold in vanilla and chocolate flavors. Since the very first start then, soymilk has never been used as a lone product although there have been attempts to do so in Brazil, initially planned to be part of the dietetic program of public schools, but it was not accepted by the students, who were more prone to consume cow milk. More recently, soymilk association with fruit juice has become increasingly popular, basically for being relatively cheap and tasteful. Therefore, this work was not designed to research soymilk itself as a viable product but, instead, its association with fruit juices, because of the commercial relevance after the economic progress of Brazil as emerging country in the world economy.

Expectable as it seems, all this consumption has led to an increased potential for dental erosion because of the bathing of the teeth in erosive acids [6–8]. Such erosive potential can be influenced by the type of drink and its pH, the amount of acid in it, the frequency and intake duration, and the calcium, phosphorous, and fluorine concentrations [9]. New techniques for detecting mineral loss in tooth structures are being researched, such as X-ray fluorescence, in order to detect early lesions and to enable the due implementation of the appropriate therapy in order to halt or revert such problem [10].

Due to the scarcity of studies that concern this subject, the aim of this study was to analyze,* in vitro*, industrialized fruit juices plus soy effects on the dental surfaces in order to measure the erosive potential of these drinks qualitatively and quantitatively.

## 2. Material and Methods

This study was approved by the University of Pernambuco Research and Ethics Committee (protocol CAAE: 0225.0.097. 000-11). Two hundred bovine incisors were treated with periodontal scaling and root planning and were polished with pumice, Robinson brush, water, and a contra-angle connected to a handpiece (KaVo®, Mod Kit Academic, Brazil). All of the teeth were stored in 0.1% (v/v) thymol solution for decontamination (4°C, 5 days). Two hundred incisors were examined under a stereo microscope (Meiji Techno, Japan) with an increase of 10x and the teeth that exhibited erosion signs, deformation, cracks, hypoplasia, or staining on the surface were excluded from the study. The final sample consisted of 70 bovine incisors.

### 2.1. Preparation of Tooth Crowns

The teeth were transversely sectioned 3 mm below the Cemento/Enamel Junction (CEJ), for the separation of the crowns accomplished with a diamond blade mounted on a cutting machine (South Bay Technology, USA). The area of coronal pulp was obliterated and filled with light cured composite resin (Advanced Concept Vigodent/Coltène, Brazil). Each crown was fixed in acrylic resin molds with wax and a dental surveyor (Olympus, São Paulo, Brazil) and wax sculpture for fixed prosthesis (Asfer, São Paulo, Brazil) so that the buccal surfaces were exposed and parallel to the resin mold to be submitted to grinding through different sandpaper grits: 400, 600, and 1200 (Arotec, São Paulo, Brazil) and polishing with a cloth soaked in 0.3-micrometer alumina (both, Arotec, St. Paulo, Brazil) in a polishing machine (APL-4 Arotec, São Paulo, Brazil) endowed with the arm to keep the parallelism necessary to microhardness and X-ray fluorescence. The crowns were cleaned with an ultrasound device (Maxiclean, Mod Unique, Brazil) in deionized water at 18.2°C MΩ·cm/25 (Direct-Q® 3 System, Millipore, France) by ten minutes and stored in a container with gauze moistened with the same water under refrigeration until the initial experimental phase took place to prevent drying.

### 2.2. pH Analysis and Acid Titration

In order to obtain the acidity degree, pH values were checked, from 15 mL of each fruit juice plus soy package, at room temperature after the opening and three days later (according to the manufacturer's instructions). A pH electrode connected to a 720 A potentiometer (Procyon of Brazil Ltda., Sao Paulo) was used, previously gauged with pH 4.0 and 7.0 buffer solutions. The samples were analyzed in duplicate. Samples that sowed pH values lower than 7, immediately after reading the hydrogen potential, were subjected to the acid titration with NaOH solution to 0.5 mol/L. The solution was slowly added by using a graduated pipette to raise the pH to neutrality (pH nearly above 7) and to analyze the volume necessary to neutralize the acidity of these beverages [11, 12]. This procedure was performed under constant stirring in magnetism (Thermix Stirrer Model 120) and the pH checkups were accomplished using an electrode connected to a 720 A potentiometer.

### 2.3. Microhardness

The microhardness was assessed by using a HMV 2000 Microdurometer (Shimadzu Corporation, Kyoto, Japan) with a penetrator/pyramidal diamond indenter Knoop type with a static load of 50 *μ*gf^2^ applied to the indenter tip remaining parallel to the surface for 30 seconds. Two parallel lines were overlaid with diamond sharp corners to determine the longest diagonal length and consequently the Knoop hardness value (KHN), which was reported directly by the machine. Each ring was subjected to three indentations distributed randomly on the surface. The measurements were recorded in an Excel spreadsheet to calculate the average of the indentations for a crown.

### 2.4. Group Allocation

The crowns were randomly allocated into seven groups with ten specimens per group, summing 70 crowns. There were six experimental groups (G1: AdeS Grape, G2: AdeS Apple, G3: Solly's Grape, G4: Solly's apple, G5: More Vita Grape, and G6: More Vita apple) and one control group constituted of distilled water (G7). The crowns were placed in individual containers with 15 mL of solution keeping the specimen fully submerged into the juices. With the aid of a stopwatch, the specimens were kept submerged under constant stirring for 15 minutes as well at 37°C and 100 rpm for thermal agitation (TE-420, Tecnal, São Paulo). Afterwards, the container was completely emptied, the liquid was discarded, and the specimen was thoroughly washed and stored with deionized water until the following cycle. This entire process was conducted twice a day (morning and evening) for 15 days [13]. All beverages used in the experiment were obtained in the same commercial setting and were equal to those observed on the manufacturing lot and validity time provided in the casing for the same solution and kept at room temperature. Only after opening were they placed on constant cooling and used for a maximum period of 3 days, as recommended by the manufacturer (Table 1).

### 2.5. Demineralization Detection Method

#### 2.5.1. Micro X-Ray Fluorescence (*μ*XRF)

**Figure 1:**
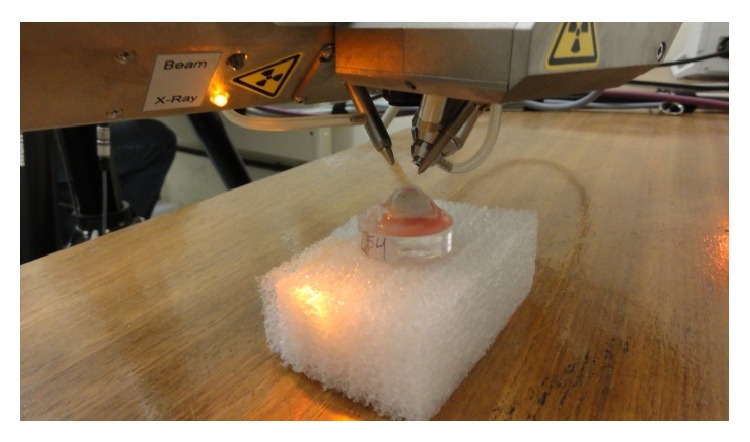
Spectrometric micro X-ray fluorescence (*μ*XRF). Bruker Model Spectra Artax (Berlin, Germany).

The enamel was subjected to chemical analysis by a portable spectrometric Micro X-ray fluorescence (*μ*XRF)—Bruker Model Spectra Artax (Berlin, Germany)—gauged with manganese (Figure 1).

An X-ray beam covers the exposed surface of tooth enamel positioned at 90° in front of the exciter source for 250 seconds, using the characteristic X-ray intensities emitted until the final spectrum is acquired. At the very moment in which two or more X-rays penetrate the detector energy pulses are read and converted to peak or two equal impulses combined. The high instrumental sensitivity allows the detector to identify the constituents of a sample to low concentrations (e.g., ppm). Through a computer program (PXRF) coupled to the apparatus, a spectrum will be drawn while there is emission and X-ray detection. This occurs repeatedly, but enough to create a visible peak, as can be observed in Figure 1 (qualitative analysis). A ruler calibration converts the peak energy position of the elements in samples of a quantitative analysis, approximate the measurement of which is given by calculating the peak area of each element. All this is done by calculating PXRF. The calcium and phosphorus amounts in the potential K*α* obtained were subsequently recorded in an Excel spreadsheet. In this study, the samples were read in triplicate before and after challenge acid. By analyzing qualitatively the same sample before and after, the content comparison of one element was proceeded, by areas overlapping of the respective peaks in the power spectrum, and also verified the new chemical presence [14, 15]. The crowns were selected by *μ*XRF analyzed to identify the present calcium ions (Ca, Z = 20) and phosphorus (P, Z = 15) located on the surface of enamel before exposure to fruit juices plus extract soybean. The potential energy emission patterns were used for the identification of Ca and P (resp., 3.691 and 2.013) and other ions.

### 2.6. Statistical Analysis

The data were statistically treated with the aid of the Software BioStat 3.0. Such data were initially submitted to test of Kolmogorov-Smirnov (Lilliefors), revealing compatible behavior with parametric patterns. Data were compared by ANOVA complemented by the Tukey's test (*p* ≤ 0.05).

## 3. Results

### 3.1. pH Analysis and Acid Titration

**Figure 2:**
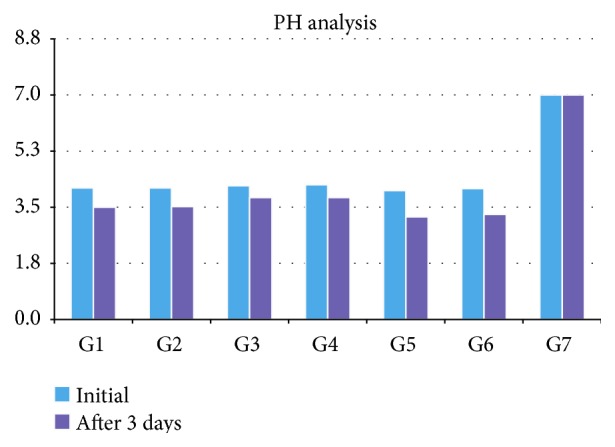
It shows the distribution of the pH values of fruit juice plus soy and the control group at baseline and after 3 days of use (groups: G1: AdeS Grape, G2: AdeS Apple, G3: Solly's Grape, G4: Solly's apple, G5: More Vita Grape, G6: More Vita apple, and G7: control). Red dashed line is the pH value for demineralization of the enamel surface (pH = 5.5).

All fruit juices plus soy extract and the control group were analyzed for pH (initial time and after 3 days) and the results are described in Figure 2.

The group that showed the lowest initial pH was G5 with 4.02, followed by G6 with 4.08. On average, the juice had an instant pH of 4.14 and 3.56 after 3 days (average decrease per day of 0.19). This variation represents a reduction of 14% compared to the initial pH, with 22%, with G5 group being the one that showed the greatest decrease, followed by G6 with 17%. The groups that showed less variation were G3 and G4, both with 9%. At baseline, fruit juices plus soymilk underwent acid titration with a solution of NaOH 0.5 mol/L. On average a solution of 1.06 was necessary to neutralize the juices. The group that needed more volume was G5 with 1.30, followed by G6 with 1.25. Both groups required approximately 1.2 times the average volume of NaOH solution 0.5 mol/L. The group that required the lowest volume was G4 with 0.70, followed by G3 with 0.80. The final pH after titration juices ranged from 7.1 to 6.96

### 3.2. Microhardness

**Figure 3:**
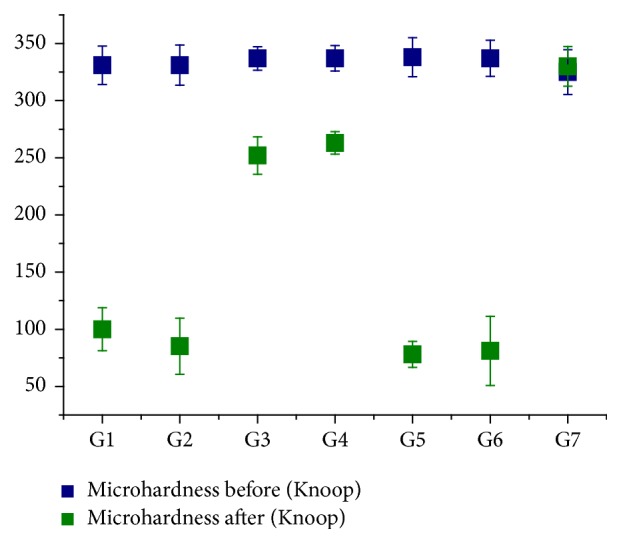
It exhibits the box plot comparing the microhardness before and after the action of fruit juice plus soy (*n* = 70). (G1: AdeS Grape, G2: AdeS Apple, G3: Solly's Grape, G4: Solly's Apple, G5: More Vita Grape, G6: Vita More apple, and G7: control; *n* = 10 per group). Statistically significant difference in all groups except the control. Paired *t*-test, G1 to G6 *p* < 0.01, G7 *p* = 0.38.

**Figure 4:**
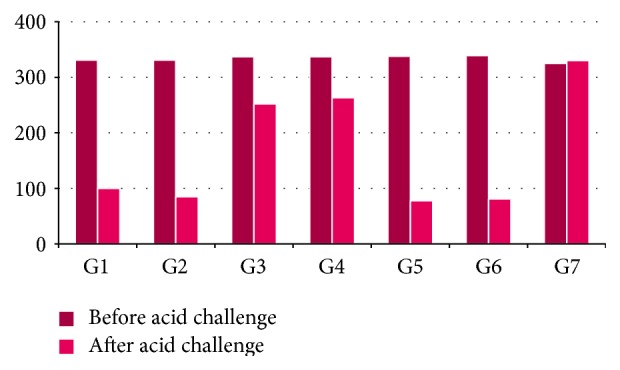
Knoop microhardness before and after acid challenge.

Figure 3 exhibits the results for microhardness; Figure 4 shows the results for Knoop microhardness. The 70 crowns showed, initially, a microhardness average of 335 (ranging from 301 to 363). The group that had the highest average microhardness was G6 with 337 ± 15.85 and the lowest was G1 with 331 ± 16.83. The Kolmogorov-Smirnov test found that all groups showed normal distribution with microhardness values (ranging from 0.139 to 0.209). The group that showed the highest microhardness was G7 with 325 ± 19.64 and the lowest was G5 with 78 ± 11.43 followed by G6 with 81 ± 30.25. Microhardness, considering all groups, was 169. The group that showed the greatest decrease was G5 (Vita More Grape) with a value of approximately 259, followed by G6 with 256 and the lowest was G7 with a value of approximately 10. The paired Student's *t*-test confirmed that, for all groups, the difference before/after was statistically significant, except for the control group. *t*-test values for groups G1 to G7 were, respectively, 26.014 (*p* < 0.01), 24.588 (*p* < 0.01), 9.762 (*p* < 0.01), 6.376 (*p* < 0.01), 38.401 (*p* < 0.01), 20.320 (*p* < 0.01), and 0.918 (*p* = 0.383).

The average decrease in microhardness between the groups was 168. G1, G2, G5, and G6 are above this average, these being the ones that decreased the microhardness after the experiment.

### 3.3. Micro X-Ray Fluorescence (*μ*XRF)

**Figure 5:**
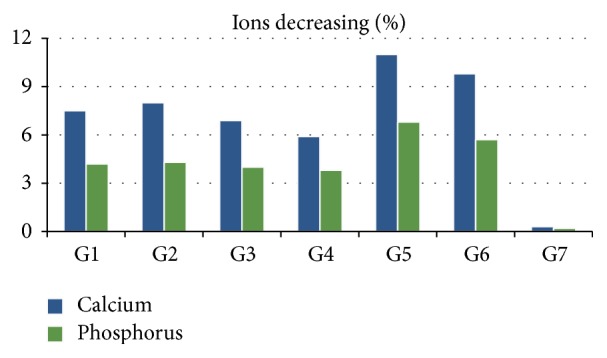
It shows the results for the ions decreasing of calcium and phosphorous after acid challenger.

The results for Ca and P are expressed in Figure 5. For all groups, the elements identified on the enamel were calcium, phosphorus, potassium (K, Z = 19), zinc (Zn, Z = 30), and strontium (Sr, Z = 38). The present Ca was 260,92 ± 2,690, P was 144,956 ± 1,1301, and the relationship between them was 1.8 ± 0.3. After exposure to fruit juice plus soy, the enamel surface was again examined by *μ*XRF. Figure 5 illustrates a comparison of the values measured for the crowns of bovine incisors, where a decrease of these ions could be observed. For all groups, except for G3, G4, and G7, initial iron elements (Fe, Z = 26) were also identified on the surface of the enamel. The total decrease (%) of Ca, P, and the ratio of Ca/P after the fruit juice action plus soy are shown in Figure 5. The mean decrease (%) was 7.06 for calcium and 4.09 for phosphorus. The groups that had the largest percentage decrease were G5 with 10.9 ± 0.4 (Ca) and 6.6 ± 0.31 (P), followed by G6 with 9.7 ± 0.21 (Ca) and 5.5 ± 0.41 (P). The group which was decreased less was G7 with 0.5 ± 0.11 (Ca) and 0.3 ± 0.081 (P). Therefore, the group that had the lowest ratio of Ca/P was G5 at 1.65 and the highest was G7 with 1.8. The average ratio of Ca/P was 1.74.

### 3.4. Statistical Analysis

The one-way ANOVA showed that this difference was statistically significant for both KHN values (*p* < 0.01), as well as the total decrease (*p* < 0.01). These results indicate that there is no statistically significant difference between the flavors of the juice brands and AdeS More Vita (*p* > 0.05). However, However, there is statistically significant difference between the brands AdeS, More Vita, and Solly's with the control group. There is a significant difference between the control group and all fruit juices plus soy.

## 4. Discussion

Fruit juice plus soy products have an enormous potential for commercial sales for the simple fact that they assemble the desirable sensory fruit characteristics with the nutritional properties of soy proteins [16]. The major question is their erosive potential concerning how much mineral structure of enamel is demineralized after their consumption.

The juices analyzed during the research are from brands available in the market, easily found and ready to be drunk by any person who stroll around in the supermarkets. What was worrying concerning the results of this work was the average for all juices pH measured at the initial time and after 3 days, with an average decrease of 14% of the initial pH showing that all juices were well below the critical value referred to in the literature [17]. G5 and G6 groups were those who had lower initial pH but also the ones that suffered the largest percentage decreases in pH, respectively, 22% and 17%. The authors emphasize that pH is one of the factors used to determine the erosive potential and that the exposure time and volume also influence erosion.

As for what concerns NaOH (0.5 mol/L) necessary to neutralize the pH of the juices, the groups G5 and G6 were those which require a greater amount of volume, respectively, 1.30 and 1.25 mL. Sodium hydroxide values were higher compared to the lower values found (G4 0.70, G3 0.80), and they are 1.2 times higher than the average found for all other groups. The chance of suffering tooth demineralization per share of these juices is real since the pH found is below the critical limit, especially for Vita More Brand. Solly's brand has a pH closer to the critical limit because of the implementation of an acidity regulator (tricalcium phosphate, 152 mg).

In this study, microhardness was used as a criteria for the dental samples in their respective groups. The goal was to use a referential greatness or physical chemistry to help in more homogeneous group formations, which could be followed throughout the experiment and serve as ballast for translational results. The study of [17] shows that the microhardness of tooth enamel human is affected by their mineral component and it may vary from 272 to 440. The crowns of the teeth showed initial microhardness values ranging from 301 to 363 (mean = 335). The largest group of average microhardness was G6 with 337 ± 15.85 and the lowest was G1 with 331 ± 16.83, both with averages close to the initial average (mean = 335). The Kolmogorov-Smirnov test showed that all microhardness values found in the groups were within normal limits. After exposure of the crowns to fruit juice plus soymilk the average decreased to 169 groups, with the largest microhardness group being G7 with 325 ± 19.64 and the lowest G5 with 78 ± 11.43 followed by G6 with 81 ± 30.25. This change represents a decrease of nearly 49% hardness values measured in juice groups. G7 showed a decrease of approximately 10.

There is a disparity between groups, where G1, G2, G5, and G6 are above the average decrease in hardness, and G3, G4, and G7 are below. These trends were confirmed by statistical tests with a statistically significant difference when comparing the results before/after, except for G7. There was no statistically significant difference between flavors of the same brand. Microhardness values measured during the study confirmed the possible erosive action identified in the analysis performed in the pH fruit juice plus soy. The X-ray fluorescence energy has been used for biological tissues, in particular inorganic materials.

A quantitative assessment by comparison can be conducted to determine the relationship between the contents of the present elements Ca and P in the mineral compound enamel [18]. The erosive fruit juice plus soymilk can be evidenced by *μ*XRF, which shows a decrease of approximately 7% and 4% of Ca P on the bovine surfaces. The groups which showed decrease of Ca were groups G5 and G6 with approximately 11% and 10% each. These results also were repeated for P, where groups G5 and G6 showed decreases of approximately 7% and 5.5%. It was observed that G7 showed a decrease of approximately 0.5% (Ca) and 0.3% (P). Quantitatively, it could also be observed that groups G5 and G6, by *μ*XRF, are above other groups and that this decrease in the present Ca and P on the surface is real. Qualitatively, the present Fe in the groups, except G3, G4, and G7, is justified by the present iron in the composed fruit juice plus soy.

However, the additional fruit juice beverage soybean, aiming to enhance their sensory properties, may cause damage due to the low pH and high acidity which may cause erosion of the enamel. Health professionals and parents should be aware about the risk for dental structures. It is important to be aware of its erosive potential to establish protocols and safer standards for consumption [1].

## Figures and Tables

**Table 1:** Composition of the juices used in the experiment according to the manufacturer's instructions.

Label	Components (200 mL portion)								
	Water	Juice	Soy extract	Sugar	Vitamins and minerals	Acidity regulator	Acidulant	Stabilizer/thickener	Others
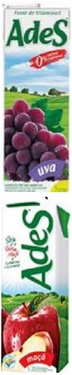	Present	Present	Present	14 g	B2; B3; B6; C; folic acid; Fe; Zn	Sodium citrate	Citric acid; maleic acid	Citrus pectin	Guar gum; calcium chloride; maltodextrin; flavoring; dye

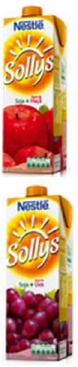	Present	Present	Present	9 g	C	Tricalcium phosphate (152 mg)	Citric acid	Polydimethylsiloxane; carrageenan	Galena gum; acacia gum; ester gum; flavoring; preservatives sodium benzoate and potassium sorbate

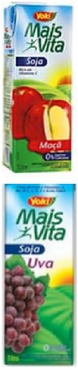	Present	Present	Present	17 g	A; B6; B12; C; D; E; folic acid; Fe	Sodium citrate	Citric acid	Pectin	Flavoring; dye
